# Integration of palliative care into South Africa’s health system: A before and after implementation study

**DOI:** 10.4102/hsag.v31i0.3242

**Published:** 2026-01-27

**Authors:** Juanita O. Arendse, Virginia Zweigenthal, Liz Gwyther

**Affiliations:** 1 Department of Public Health and Family Medicine, Faculty of Health Sciences, University of Cape Town, Cape Town, South Africa; 2 Western Cape Department of Health and Wellness, Cape Town, South Africa; 3 Department of Family, Community and Emergency Care, Faculty of Health Sciences, University of Cape Town, Cape Town, South Africa

**Keywords:** integration, palliative care, policy adoption, healthcare professionals, morphine usage, low- and middle-income countries, primary healthcare services, service delivery evaluation

## Abstract

**Background:**

Providing palliative care (PC) is an ethical obligation, and health systems must ensure access for all patients with life-limiting and life-threatening conditions. South Africa’s National Policy Framework and Strategy for Palliative Care mandates integration into public services.

**Aim:**

To assess integration of PC services, previously neglected, before and 2 years after policy implementation.

**Setting:**

South Africa’s Cape Metro District.

**Methods:**

A cross-sectional descriptive study compared baseline (March 2019–June 2019) and follow-up (March 2021–June 2021) via desktop and document review. Fifteen documents and quantitative changes in deidentified morphine use, as a tracer for outpatient PC pain control, and International Classification of Diseases (ICD)-10 coding, as a proxy for PC service availability, were reviewed. Other key measures included financing (number of beds, funding per bed), planning and service delivery (care pathways, referral process).

**Results:**

Palliative care was partially integrated into primary care. Indicators of policy adoption included an increased number of PC beds in intermediate care facilities, significant growth in healthcare professionals trained in PC and increased morphine usage in some sub-districts. However, integration remained uneven across the district.

**Conclusion:**

Integrating PC into an already overburdened health system is challenging. Further integration of PC is needed, together with training tailored for diverse professionals. Effective practices in some subdistricts of the region, such as optimised morphine prescribing and streamlined referral pathways, could serve as models for subdistricts where integration is less advanced.

**Contribution:**

The study documents positive changes following policy implementation and underscores the importance of strong project management and resource mobilisation for successful integration of complex services like PC.

## Introduction

National health systems are responsible for ensuring that palliative care (PC) services are included in the continuum of care for patients with serious life-limiting and life-threatening conditions (World Health Organization [WHO] 2016). Providing PC is also an ethical obligation for all health professionals, with such care accessible across the care pathway (Arendse 2023). The WHO (2021) considers governance and leadership as critical to ensure the development of appropriate national PC policy.

Globally and in South Africa, PC has been delivered by non-governmental organisations (NGOs) such as hospices. Gwyther et al. (2018) found that only 17.8% of South African patients requiring PC services had access, and NGOs were unable to meet the total need for PC in the country. However, in South Africa in 2017, the National Policy Framework and Strategy for Palliative Care (National Department of Health, South Africa 2017) prescribed integration of services into the public sector healthcare system, expecting each province to develop implementation plans to integrate PC into their health system. Accordingly, the Western Cape Provincial Health and Wellness Department (WCDHW) initiated a PC service prior to the coronavirus disease 2019 (COVID-19) pandemic, involving no additional resources. Health system challenges identified by the National Policy Framework and Strategy for Palliative Care (NPFSPC) included service delivery gaps regarding spiritual care, poorly defined care pathways for PC patients (including inadequate communication and referral processes) and shortage of appropriately skilled personnel (Arendse 2023). Further challenges identified included inadequate knowledge of the concept of PC by several actors, and gaps in the community-based service-delivery platform because of limited PC training of contracted lay community health workers (CHWs; National Department of Health, South Africa 2017). The NPFSPC guides PC service implementation for life-threatening and life-limiting conditions and includes clinical guidelines and tools for provinces to plan, implement and monitor PC services by building on existing services and policy priorities. It strengthens the district-based health system, linking health facilities and community-based services to provide patient-centred care, and draws on the strong national network of hospices and NGOs that have led the provision of care to patients and families and trained healthcare providers.

Although information in low- and middle-income countries (LMICs) on the cost effectiveness of PC service delivery is scant, Krakauer et al. (2021) suggest that the return on investment from the initial costs for policy development, essential medicine procurement, education and training and additional staff yields returns over time. Savings come from the reduction in unnecessary admissions and invasive interventions, improving the quality of life of the patient without shortening it, protecting the patient and family from impoverishment and improving the satisfaction of the family caregivers. Continuity of care is dependent on carefully designed, defined care pathways and referral processes. For instance, O’Connor et al. (2016) described an Australian model where nurse practitioners facilitate inter-professional liaison between levels of care while ensuring continuity of care for patients in community settings. Morey et al. (2021) found that continuity of care involved consistency in both information exchange between care providers across the care pathway and consistency of treatment while adapting care plans to patients’ needs. Such relational continuity between patient and provider at the level of care is fundamental and particularly important near the end of life.

Palliative care services proposed in the NPFSPC include home-based care and support services to patients and their families (National Department of Health, South Africa 2017; Western Cape Government Health 2018) based on the surmised preferred place of death being at home (Powell et al. 2014) and using the opportunity to build support around families in preparation for a home death. Hongoro and Dinat (2011) argued that in South Africa, a hospital-based outreach programme is more cost-effective than repetitive admissions to already burdened and overcrowded hospitals and improves the quality of life of the patient and family in the home setting. An outreach visit amounted to 50% of the cost of one patient-day equivalent in a district hospital bed. Furthermore, Daviaud et al. (2018) found that the cost of home-based PC offered by CHWs in South Africa was 10% of the cost of in-patient PC over a 2-week period. Offering support to patients and their families in the form of outpatient hospital-based services reduces admissions and increases the rate of home deaths, offering a feasible and cost-effective model (DesRosiers et al. 2014).

The NPFSPC argues for integration of PC into the health system, and that integration of a new intervention can occur at any level(s) of the health system (local, district, regional or national) in relation to critical health system functions (National Department of Health, South Africa 2017). Atun et al.’s (2010) framework can be used to determine the extent of integration of an intervention into the general health system and can be assessed as full, partial or no integration. ‘No integration’ refers to no evidence of any form of integration according to the elements contained in the framework. ‘Partial’ and ‘full’ integration are determined with due consideration for the elements contained in the framework and are triangulated with qualitative information gained through interviews (Atun et al. 2010). The framework identifies the functions of finance, planning, service delivery, monitoring and evaluation, governance and demand generation. This study describes PC services offered in the Cape Metro District, South Africa, at the onset of the implementation of the NPFSPC in early 2019 compared with 24 months post-implementation in 2021.

## Research methods and design

### Design

The cross-sectional descriptive study describes the PC services at baseline in March 2019–June 2019 and 24 months later (March 2021–June 2021) following implementation of the NPFSPC, to assess the integration of PC into the Cape Metro District healthcare system. Atun et al.’s (2010) conceptual framework for analysing integration of targeted health interventions into health systems was employed with a focus on the health system characteristics of financing, capacity planning, service delivery (care pathways, referral process and levels of care) and monitoring and evaluation (PC cascade – an algorithm that indicates probable PC need, headcounts and access to morphine).

### Study context

The study took place in Cape Town in the Western Cape province of South Africa. Known as the Cape Metro Health District and consisting of eight sub-districts that make up four sub-structures (SS1–SS4), the region is affected by a quadruple burden of disease that includes chronic diseases, communicable diseases such as human immunodeficiency virus (HIV)/acquired immunodeficiency syndrome (AIDS) and tuberculosis (TB), injuries and maternal and neonatal diseases because of poverty (Gwyther 2002). The Cape Metro Health system forms part of the provincial health system and comprises tertiary and central, secondary or regional, district and specialised hospitals and primary healthcare facilities including municipal health services. Established stakeholder relationships exist between the provincial health system and government-funded NGOs, Higher Education Institutions, municipal health services and the private healthcare sector. The WCDHW department implemented the NPFSPC within existing resources and governance responsibilities rather than with dedicated staff, governance and resources (Arendse 2023). This approach aimed to encourage the integration of PC services into the public sector health system, moving away from offering PC as a separate service.

### Data collection and analysis

**FIGURE 1:**
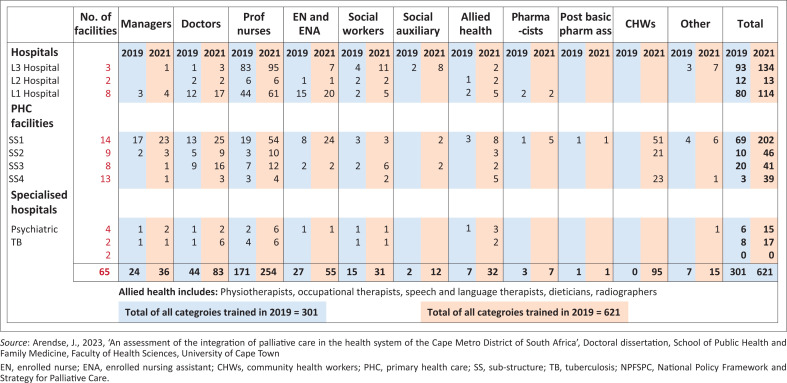
Palliative care training at baseline versus 24 months post-NPFSPC implementation.

A desktop review and document analysis were conducted at baseline and 24 months after implementation of the policy. Analysis of documents is a recognised qualitative analysis method to examine the response to policy (Bowen 2009; Dalglish, Khalid & McMahon 2020). Fifteen WCDHW policy and other documents that pertain to PC service provision were included and systematically reviewed using the READ approach (Dalglish et al. 2020). Sections of pertinent information were inductively retrieved, organised and written-up. Guided by the research question – ‘To what extent was PC integrated into the health system?’ – and content analysis was used to code and organise information into relevant categories. Reliability and validity of the coding and categorisation were checked with the study supervisors to ensure consistency over time. A review of quantitative changes in morphine prescribing and access and PC service availability based on ICD-10 coding was conducted and captured in a spreadsheet. Figure 1 summarises the main aspects investigated in the Atun et al. (2010) framework, outcome measures and the data sources.

### Ethical considerations

Ethics approval was obtained from the University of Cape Town ethics committee (HREC: 058/219), and permission was given by the WCDHW to conduct fieldwork among staff and patients at the study sites and to access data related to morphine drug prescribing, PC cascade indicators and NGO datasets related to PC in the district (Arendse 2023). The Provincial Health Data Centre provided datasets and information required at baseline and 24 months after NPFSPC implementation, including deidentified aggregated information related to ICD-10 codes for diseases for PC and morphine prescribing, supply and health facility access points.

## Results

### Finance: Number of beds, funding and types of services

**FIGURE 2:**
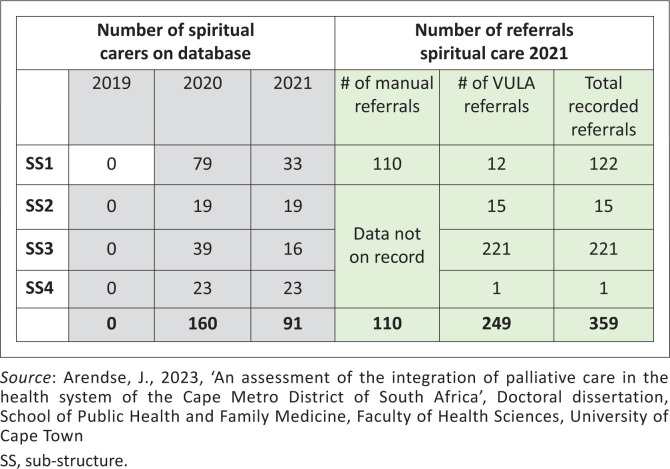
Sub-structure reports and VULA app data set on spiritual carer access from mid-2020 to mid-2021.

In 2019, the nine intermediate care facilities (ICFs) in the Cape Metro District were funded from ZAR471 to ZAR680 per bed per day for the provision of sub-acute in-patient services rendered by NGOs. The range reflects variations in the rehabilitation services offered at each ICF and the difference in the number of beds per geographic area. By 2021, the funding norm per bed per day had increased between 11.5% and 17% to between ZAR525 and ZAR793 per bed per day. The number of ICF beds in SS3 increased from 187 to 237 for 8 months to accommodate the COVID-19 pandemic in-patient PC needs. Figure 2 summarises the funded ICFs in the metro district from 2019 to 2021.

The number of ICF beds expanded by 17.0%, from 564 in 2019 to 644 in 2020 (to accommodate the COVID-19 pandemic), and although this reduced to 594 in 2021, it remained 5.9% higher than baseline in 2019. Since 2019, capacity for PC in-patient care expanded in three of the four SSs increasing by between 5.85% and 17%.

### Planning: Healthcare professionals trained in palliative care

**FIGURE 3:**
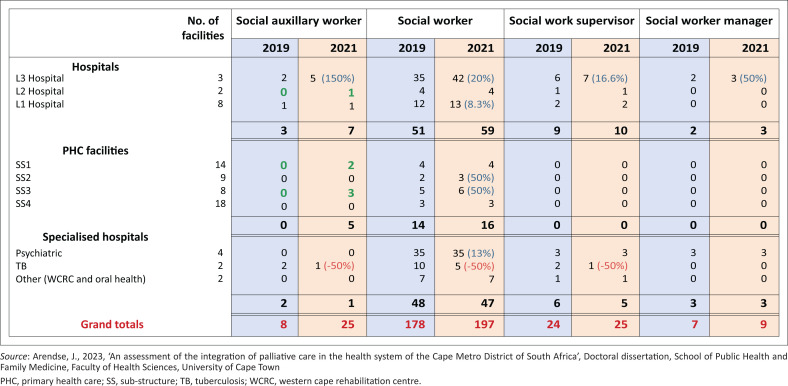
Social worker categories employed across the Cape Metro District at baseline and 24 months later.

Training in the 40-hr introduction to PC course in SS1 increased by 192% (*N* = 202), compared to the other three sub-structures that increased, but with numerators below 50. Despite the interruption in training because of the COVID-19 pandemic, 301 (1.5%) of 20,691 district health professionals were trained by 2019. By 2021, this had increased to 621 (2.9%) of the 21,382 employed health professionals, a statistically significant increase (χ^2^ = 18 600; *p* = 0.00). Overall, there were 106.3% more trained health professionals, with 48.5% more professional nurses, 103.7% more enrolled nurses, 88.6% more doctors and 153% more social workers who completed the training. The baseline of trained allied health professional was low at seven individuals, which increased by 357.0%. Training for CHWs only commenced after baseline in 2019, resulting in an increase from 0 to 95 CHWs trained by 2021. Figure 3 summarises the health professionals in the district trained in PC, showing statistically significant increases (*p* < 0.05) in all categories from 2019 and 2021. The proportion of all employed social workers trained in PC increased significantly from 7.2% in 2019 to 13.4% in 2021 (χ^2^ = 39.2; *p* < 0.0001), but the proportion of social auxiliary workers trained increased from 25.0% to 48.0%, which is not statistically different. Figure 1 provides further details about PC training at different levels of healthcare.

### Service delivery: App-based referrals, social workers and multidisciplinary teams

The VULA mobile phone referral computer application, used across the WCDHW, enables consultation with specialists and referral of patients. Focusing on one pilot site used for PC referrals, Mitchell’s Plain District Hospital showed a steady increase in patient consultations and referrals using VULA: In 2019, there were 34 referrals, growing to 497 in 2021, reflecting a 794% increase in 2020 and a further 63% in 2021. Patients were often referred for more than one reason, with 77% of patients requiring referral for continuity of care, while 67% required pain and symptom control as well as psychosocial support, and 26% required spiritual care. Figure 2 shows the number of spiritual carers and referrals for spiritual care. While many patients had more than one diagnosis, the lead presenting diagnosis was cancer (35.6%), followed by kidney failure (14.5%), heart disease (12.7%), cerebrovascular accidents (11.7%) and dementia (10.8%).

**FIGURE 4:**
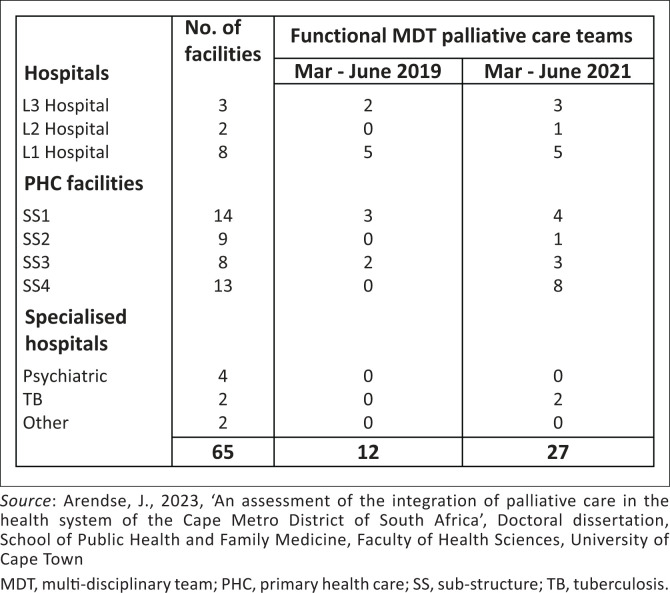
Functional palliative care teams at baseline versus 24 months later in the Cape Metro District.

Figure 3 shows the increase in the number of social workers and auxiliary social workers, essential to enable comprehensive PC, employed in the region. The functional multi-disciplinary teams (MDTs) comprising groups of health professionals collaborating for holistic provision of PC at primary health care (PHC) facilities increased to 36%. Figure 4 reflects the comparison between 2019 and 2021. Although there was a statistically significant increase – 56% (χ^2^ = 8.2; *p* = 0.0042) in the number of functional MDTs, 58% of facilities still lacked functional PC MDTs.

### Monitoring and evaluation: Morphine usage data

Morphine dispensing was used as a proxy indicator for provision of PC because, in the facilities evaluated, it is largely dispensed for pain control, common in PC-related diagnoses. In primary care, most morphine is used for pain control in contrast to hospital usage where morphine may be mainly used for post-surgery pain control. Figure 4 shows that the highest increases in overall morphine usage were within the Mitchells Plain and Klipfontein subdistricts – increases of 19% and 15%, respectively. Compared to all prescribed medications, between 2019 and 2021, there were statistically significant increases in the proportion of morphine prescribing and dispensing in all sub-districts.

### Monitoring and evaluation: ICD-10 codes

In 2019, the PC ICD-10 coding (Z51.5, the encounter for PC) from the WCDHW web-based dashboard, known as the Single Patient Viewer, reflected 98 health service encounters (excluding central hospitals). By 2021, the frequency of this ICD-10 code had notably increased (Figure 1).

## Discussion

Palliative care forms part of the management of a range of life-threatening conditions and the life-limiting stages in the natural course of a diverse set of chronic disease conditions. To address this critical service gap, it is important to assess the extent of integration of PC services in existing health services.

The number of ICF beds increased over the study period, along with funding per bed. By 2021, PC – with clear admission criteria (Peters 2020) – was included in the service packages offered at ICFs in three of the four SSs and had expanded by between 5% and 17% since baseline. A Canadian study (Zimmermann et al. 2006) supports the importance of clear admission criteria to in-patient facilities to ensure appropriate lengths of stay. Significant progress was made in training healthcare professionals in PC, and over the 24 months, the number of healthcare providers had doubled. Despite the huge uptake in basic training, local training need analyses were difficult to project in the absence of a skills framework that would provide a training baseline. Certain job functions require specific training, and employees should be assessed to determine their training needs. Large-scale PC training requires consideration of the methods, learner location and circumstances, class size, course length, content and teaching methods to accommodate the needs of PC teams. A Canadian study demonstrated that interprofessional approaches are essential to effect large-scale training in PC (Pereira et al. 2021). Furthermore, we showed a significant increase in the recruitment and appointment of all social work categories and an increase in public sector health facilities that have functional MDTs, essential for comprehensive PC.

There are no standard referral mechanisms in the WCDHW although context-specific mechanisms such as the VULA app have been explored. The WHO’s (2016) guide for programme managers implementing PC advocates that national health systems should ensure that referral mechanisms are in place, including for PC patients across the care continuum – a gap that remains in the Western Cape and requires attention.

South Africa, together with other African and LMIC countries, has an inadequate use of morphine (Walther Global Palliative Care & Supportive Oncology 2023), and Hofmeyr and Phelanyane (2021) describe lack of confidence in prescribing morphine at lower levels of care in South Africa. Indeed in 2020, South Africa’s consumption compared poorly to the UK, at 28 mg/person compared to 55 mg/person (Walther Global Palliative Care & Supportive Oncology 2023). However, consumption has increased since 2013, particularly between 2018 and 2020 when it increased four-fold, from 7 mg/person in 2018. This study also found an increase in morphine dispensing in some subdistricts, showing how actively the policy is being implemented, benefiting patients in these areas. Lower morphine utilisation facilities can learn from higher-performing facilities by comparing their practices and exploring how these successes can be implemented within their context. The 3% decrease in overall morphine use between 2019 and 2021 may directly reflect the COVID-19 pandemic as services and resources were redistributed and de-escalated because of the overwhelming platform response required to treat patients with COVID-19. Nonetheless, appropriate management of pain is inadequate, but this is not unique to LMICs. For example, Nguyen et al. (2023) also report these disparities in the United States among vulnerable populations, the elderly and rural communities.

By 2021, the PC ICD-10 code had notably increased when compared to 2019. However, based on PHC morphine usage, PC need may be under-reported because these counts changed minimally when central hospitals were included or excluded. Underreporting is not unique to the Cape Metro District. In Thailand, researchers found that even though patients had access to good PC, the use of ICD-10 codes was low (Fumaneeshoat 2018). ICD-10 codes for diseases that indicate PC disease consultations or admissions (‘episodes’) will feed into the data centres’ PC cascade (the algorithm indicating probable PC need), which is still in development. The PC cascade may include some ICD-10 codes for TB, HIV, cancers, mental health, cardiovascular disease and chronic kidney disease.

The Atun et al. (2010) conceptual framework, used to structure the outcome measures in this study and assess policy integration, must be viewed within the broader context because changes in a health system can affect the broader context, and similarly changes in the broader context can influence the health system.

### Limitations

Limitations for this study include the assumption that the workforce in the Cape Metro District was stable over the 2-year period. The proportions of staff trained in PC may be affected by this assumption and therefore the validity of the statistical tests that we used to determine change. Furthermore, it may be too early to tell whether PC initiatives are sustainable and to determine the exact extent of integration.

### Recommendations

Training for already qualified health care workers in the introduction to PC course needs to be scaled up. This requires engagement with higher education institutions, motivating for the inclusion of PC in the curriculum for undergraduate training of health and allied health care professionals. In addition, the curriculum for the training of CHWs needs to include the provision of PC in the home setting, as well as how to support the PC patients’ caregivers. In provincial health systems, a framework for spiritual care that includes a database for the entire health system needs to be formulated.

## Conclusion

Introducing a new service into an already over-burdened health system is not easy, and the scale and complexity of a new service must be considered meticulously for success (WHO 2007). This study, recognising the complexity, assessed the integration of PC into routine health services in the Cape Metro District, demonstrating positive changes and integration in a range of areas, including the number of PC beds, training, human resources, PC referrals and morphine usage, indicating a move from no integration to partial integration of PC services into primary care services. These findings were impacted by the COVID-19 pandemic, which occurred during the 2-year study period. The pandemic most likely promoted and highlighted the need for PC but also created significant challenges such as access to pain medication and healthcare services for non-COVID-19 and non-emergency needs. Despite resource constraints and an over-burdened health system, strong project management can mobilise resources for successful integration of a complex service such as PC towards the realisation of access to PC as a human right.
